# Key Molecules and Pathways Underlying Sporadic Amyotrophic Lateral Sclerosis: Integrated Analysis on Gene Expression Profiles of Motor Neurons

**DOI:** 10.3389/fgene.2020.578143

**Published:** 2020-11-09

**Authors:** Jianing Lin, Pian Huang, Weineng Chen, Chenghui Ye, Huanxing Su, Xiaoli Yao

**Affiliations:** ^1^Department of Neurology, The First Affiliated Hospital, Sun Yat-sen University, Guangdong Provincial Key Laboratory of Diagnosis and Treatment of Major Neurological Diseases, National Key Clinical Department and Key Discipline of Neurology, Guangzhou, China; ^2^Department of Neurology, The Seventh Affiliated Hospital of Sun Yat-sen University, Shenzhen, China; ^3^State Key Laboratory of Quality Research in Chinese Medicine, Institute of Chinese Medical Sciences, University of Macau, Macao, China

**Keywords:** sporadic amyotrophic lateral sclerosis, gene expression profiles, motor neurons, bioinformatics analysis, *FN1* gene

## Abstract

Amyotrophic lateral sclerosis (ALS) is a neurodegenerative disorder characterized by progressive loss of motor neurons. The complex mechanisms underlying ALS are yet to be elucidated, while the lack of disease biomarkers and therapeutic options are associated with the poor prognosis of ALS patients. In this study, we performed bioinformatics analysis to clarify potential mechanisms in sporadic ALS (sALS). We compared three gene expression profiles (GSE18920, GSE56500, and GSE68605) of motor neurons obtained from sALS patients and healthy controls to discover differentially expressed genes (DEGs), and then performed integrated bioinformatics analyses to identify key molecules and pathways underlying sALS. We found that these DEGs were mainly enriched in the structure and functions of extracellular matrix (ECM), while functional enrichment in blood vessel morphogenesis was less correlated with motor neurons. The clustered subnetworks of the constructed protein-protein interaction network for DEGs and the group of selected hub genes were more significantly involved in the organization of collagen-containing ECM. The transcriptional factors database proposed RelA and NF-κB1 from NF-κB family as the key regulators of these hub genes. These results mainly demonstrated the alternations in ECM-related gene expression in motor neurons and suggested the role of NF-κB regulatory pathway in the pathogenesis of sALS.

## Introduction

Amyotrophic lateral sclerosis (ALS) is a fatal disease characterized by notable degeneration in upper and lower motor neurons (Oskarsson et al., 2018). The clinical features of ALS vary in upper and lower motor neuron signs in different regions, and some patients may develop cognitive and behavioral symptoms (van Es et al., 2017; Oskarsson et al., 2018). The incidence of ALS rises in midlife and reaches the peak at the age of 70 years or above, while the prevalence has been estimated at about 4–8 in 100,000 people in most populations (Marin et al., 2018; Longinetti and Fang, 2019). Progressive weakness in muscles will lead to death eventually within 2–3 years after disease onset, mainly due to respiratory failure (Oskarsson et al., 2018). Apart from riluzole and edaravone as two widely FDA-approved drugs that can prolong the survival time for several months, ALS is still an incurable disease with limited therapeutic options (Brown and Al-Chalabi, 2017).

It is essential to explore the underlying mechanisms in ALS, but the etiology of ALS remains largely unknown. Both genetic and environmental factors play crucial roles in the pathogenesis of ALS. Since the first mutation in Cu/Zn superoxide dismutase (SOD1) gene was discovered, more than 25 ALS-related genes have been identified to date, due to the development of high-throughput DNA sequencing (Chia et al., 2018; Nguyen et al., 2018). More than 90% of ALS cases are sporadic ALS (sALS) without reported family history, while two-thirds of familial ALS (fALS) are hereditary (Renton et al., 2014). Mutations in common ALS-related genes, including SOD1, TARDBP, C9ORF72, and FUS, were found in less than 50% fALS and about 5% sALS, and genetic factors were found in only two thirds of fALS and about 10% of sALS, indicating that genetic factors cannot fully explain the pathogenesis of ALS, especially the sporadic form of ALS (Renton et al., 2014; Zou et al., 2017). Vigorous physical activity, smoking and exposure to pesticides, heavy metals, and electromagnetic fields were suspected risk factors for ALS (Zufiria et al., 2016).

The development in microarray and sequencing technology makes it possible for researchers to study diseases at DNA and RNA levels, and comprehensive analyses of multiple studies are more reliable and effective in searching for common gene targets (Kotni et al., 2016; Al-Chalabi et al., 2017). Hence, we performed integrated bioinformatics analysis among three gene expression series (GSE) from the Gene Expression Omnibus (GEO) to identify differentially expressed genes (DEGs) in motor neurons between sALS cases and healthy controls (HC). Subsequently, we conducted functional and pathway enrichment for DEGs and explored their protein-protein interactions (PPI), followed by identification of hub genes and clustered subnetworks from the PPI network, and the discovery of regulatory transcriptional factors (TFs). Our study aims to provide new insights into the pathogenesis of sALS and clues in discovering novel biomarkers and therapeutic options.

## Materials and Methods

### Data Collection and Processing

According to the main purpose of our study, we searched relevant gene expression profiles from the GEO database^1^, a public functional genomics data repository. Datasets focusing on familial ALS, *in vitro* studies, or studies concerning samples of motor cortex and spinal cord without the isolation of motor neurons were excluded in our analysis. At last, we collected three gene expression profiles: GSE18920, GSE56500, and GSE68605. Among them, GSE18920 and GSE56500 were based on GPL5188 platform ([HuEx-1_0-st] Affymetrix Human Exon 1.0 ST Array), while GSE68605 was based on GPL570 platform ([HG-133_Plus_2] Affymetrix Human Genome U133 Plus 2.0 Array). The original researchers obtained motor neurons through laser capture microdissection in human spinal cords, followed by extraction of total RNA and gene expression profiling with Affymatrix microarrays. Gene expression profiles of 19 neurologically HC and 19 sALS patients were included, and we excluded fALS patients in our analysis. As all gene expression profiles from GEO were public online, we did not perform any experiments on human tissues in our study. The overview of detailed information of three gene expression series selected in our study was shown in Table 1.

**TABLE 1:** Details of three gene expression profiles derived from GEO database.

**Series**	**GSE18920**	**GSE56500**	**GSE68605**
Platform	GPL5188	GPL5188	GPL570
Array	HuEx-1_0-st; Affymetrix Human Exon 1.0 ST Array	HuEx-1_0-st; Affymetrix Human Exon 1.0 ST Array	HG-133_Plus_2; Affymetrix Human Genome U133 Plus 2.0 Array
Tissue	Motor neurons from lumber spinal cord	Motor neurons from cervical spinal cord	Motor neurons from cervical spinal cord
**Healthy control**	10	6	3
Male: Female	8:2	5:1	1:2
Mean age (years)	72.8	61.7	60.0
**Sporadic ALS**	12	3	4
Male: Female	6:6	2:1	1:3
Mean Age (years)	66.4	65.7	60.5
Mean ALS duration (years)	2.88	Not available	2.27
Bulbar onset: Spinal onset	6:6	Not available	2:2

### Identification and Analysis of DEGs

GEO2R^2^ was an online analysis tool to compare two or more groups of samples for DEGs by employing GEOquery and limma R packages from the Bioconductor project^3^. Here we utilized GEO2R to discover DEGs between sALS and HC, and Graphpad Prism 8 (GraphPad Software Inc., San Diego, CA, United States) was used for volcano plot visualization. The cut-off criteria were set at *P* < 0.05 and | log_2_Fold Change (FC)| > 1 for DEGs. An online Venn diagram was applied to discover the overlapped DEGs from all gene expression profiles^4^.

### Functional and Pathway Enrichment Analysis of DEGs

We performed function and pathway enrichment analysis based on Metascape^5^, an integrated online tool for gene list annotation and biological analysis (Zhou et al., 2019). Gene ontology (GO) analysis is a common method in functional enrichment analysis, aiming to provide gene annotations in biological processes (BP), molecular functions (MF), and cellular components (CC). Kyoto Encyclopedia of Genes and Genomes (KEGG) database is crucial in understanding high-level functions and utilities of biological systems, which is especially famous for its pathway enrichment analysis. The overlapped DEGs was analyzed and visualized by Metascape with the criteria of minimum overlap > 3, *P*-value cut-off < 0.01, and minimum enrichment score > 1.5.

### PPI Network Construction and Identification of Hub Genes

The Search Tool for the Retrieval of Interacting Genes (STRING) online database (version 11.0)^6^ is a common tool for predicting PPI networks with increased coverage in genome-wide experimental datasets (Szklarczyk et al., 2019). The predicted PPIs of DEGs was under the criteria at a medium confidence (minimum required interaction score > 0.4) and subsequently visualized by Cytoscape software (version 3.7.2)^7^, an open source software for visualization and integration of biomolecular interaction networks (Shannon et al., 2003). The Molecular Complex Detection (MCODE) plugin (version 1.6.1) was applied to discover highly connected clusters in a given network, with the analysis criteria as degree cutoff = 2, node score cutoff = 0.2, K-core = 2, and maximum depth = 100. The key nodes were selected according to the scoring of maximum correlation criterion (MCC) by using the cytoHubba plugin, which explores important nodes and subnetworks by topological algorithms. Twenty genes scoring the highest were identified as hub genes in our study.

### Analysis of Hub Genes and the Regulatory Transcriptional Network

The identified clusters with more than five nodes and the selected hub genes in our study were analyzed by Metascape, respectively, for enrichment analysis. The ClueGO (version 2.5.7) and CluePedia (version 1.5.7) plugins in Cytoscape were applied for visualization of the functionally grouped networks of the hub genes. The Transcriptional Regulatory Relationships Unraveled by Sentence-based Text mining (TRRUST) (version 2)^8^ is a manual database of human and mouse transcriptional regulatory networks, which was utilized to discover key regulators for the hub genes in our study (Han et al., 2018). Depending on the recorded TFs-targeted genes regulatory relationships in TRRUST, we identified TFs that regulated the expression of hub genes and then visualized the regulatory network by STRING.

## Results

### Identification of DEGs in ALS

**FIGURE 1:**
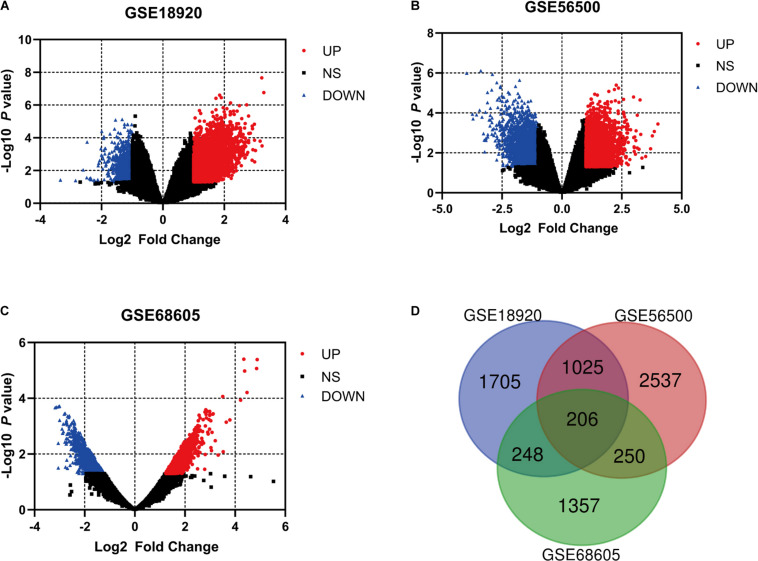
The analysis of differentially expressed genes (DEGs) of multiple gene expression profiles by GEO2R. **(A–C)** The volcano plots of the distribution of multiple gene expression series, with red dots and blue dots indicating upregulated and downregulated genes, respectively. **(D)** Online Venn diagram tool was applied to discover the overlapping DEGs of three datasets.

After the exclusion of fALS samples, three selected gene expression profiles (GSE18920, GSE56500, and GSE68605) were analyzed in our study. With the standardization of the value of gene expression and calculation by GEO2R tools, the DEGs were identified between sALS patients and HC of each series according to the filtering criteria, which were displayed in the volcano plots respectively (Figures 1A–C). A total of 3,184 DEGs were found in GSE18920, with 2,428 upregulated and 756 downregulated. In GSE56500, 4018 DEGs were found, including 2,100 upregulated and 1,918 downregulated genes. Of 2,061 DEGs found in GSE68605, 971 were upregulated and 1,090 were downregulated. In total, 206 overlapping DEGs were identified by Venn diagram and then processed for further analysis (Figure 1D).

### Function and Pathway Enrichment Analysis of DEGs

**FIGURE 2:**
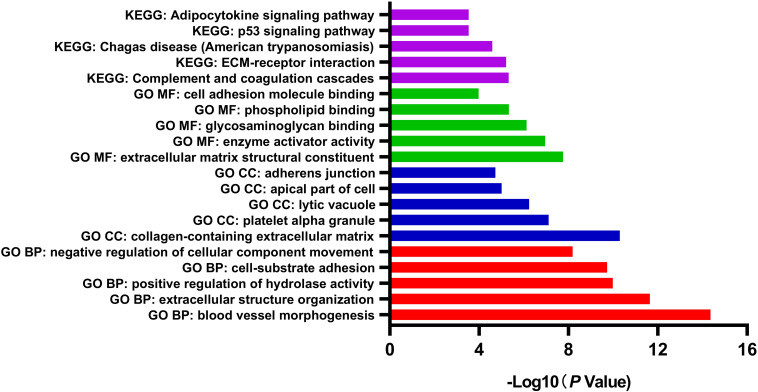
Gene Ontology (GO) function and pathway enrichment analysis of all differentially expressed genes by Metascape. The results were colored by different GO terms.

GO and pathway enrichment analysis of DEGs was performed by Metascape, which is useful in identifying crucial biological functions of a specific gene group. The results showed that DEGs were significantly enriched in various biological processes, including blood vessel morphogenesis, extracellular structure organization, positive regulation of hydrolase activity, and cell-substrate adhesion. DEGs were more likely to be enriched in collagen-containing extracellular matrix (ECM), platelet alpha granule, lytic vacuole, apical part of cell, and adherens junction in cellular components subgroup. In terms of molecular functions, DEGs were involved in several functions, including ECM structural constituent, enzyme activator activity, glycosaminoglycan binding, phospholipid binding, and cell adhesion molecule binding. Referring to the KEGG database, the analysis revealed enrichment mainly in pathways of complement and coagulation cascades, and ECM-receptor interaction (Figure 2). Considering the object of this study as motor neurons, the comprehensive enrichment analysis indicated crucial molecular alternation in ECM structural organization and relevant functions in sALS motor neurons.

### PPI Network Construction and Identification of Subnetworks

**FIGURE 3:**
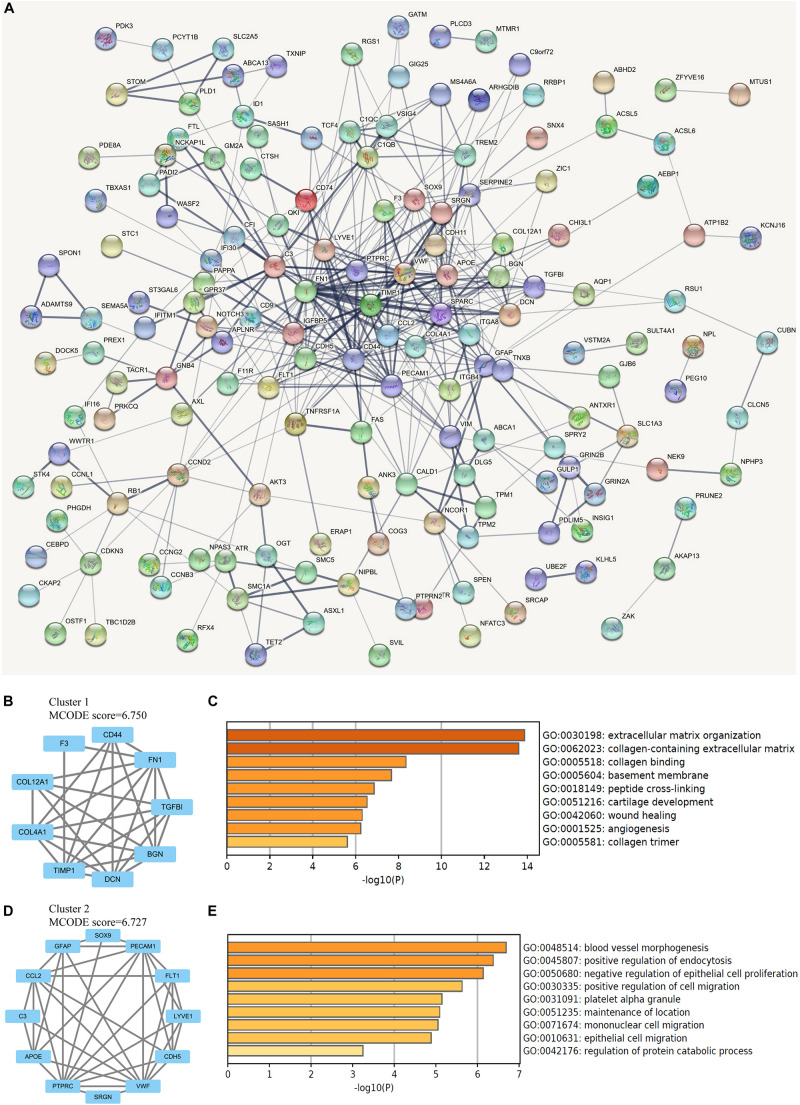
Network analysis of differentially expressed genes (DEGs). **(A)** The protein-protein interaction (PPI) network of overlapping DEGs, in which thicker line indicates stronger data support. **(B)** The clustered subnetwork 1 identified from the whole PPI network. **(C)** Enrichment analysis of the cluster 1 by Metascape. **(D)** The clustered subnetwork 2 identified from the whole PPI network. **(E)** Enrichment analysis of the cluster 2 by Metascape.

The protein interactions among all 206 DEGs was analyzed by STRING online database. With the confidence at medium level, the PPI network was constructed with an average node degree of 4.03 and the PPI enrichment *P* < 1.0 × 10^–16^ (Figure 3A). The obtained files were subsequently managed by Cytoscape for visualization. The analysis by MCODE plugin revealed several subnetworks in the whole DEGs network. However, only two subnetworks containing more than five nodes were identified (Figures 3B,D). The subnetwork with the highest MCODE score was significantly enriched in ECM organization, collagen-containing ECM, collagen binding, and basement membrane, while the other one was enriched in blood vessel morphogenesis, positive regulation of endocytosis, and negative regulation of epithelial cell proliferation (Figures 3C,E).

### Identification of Hub Genes and Enrichment Analysis

**FIGURE 4:**
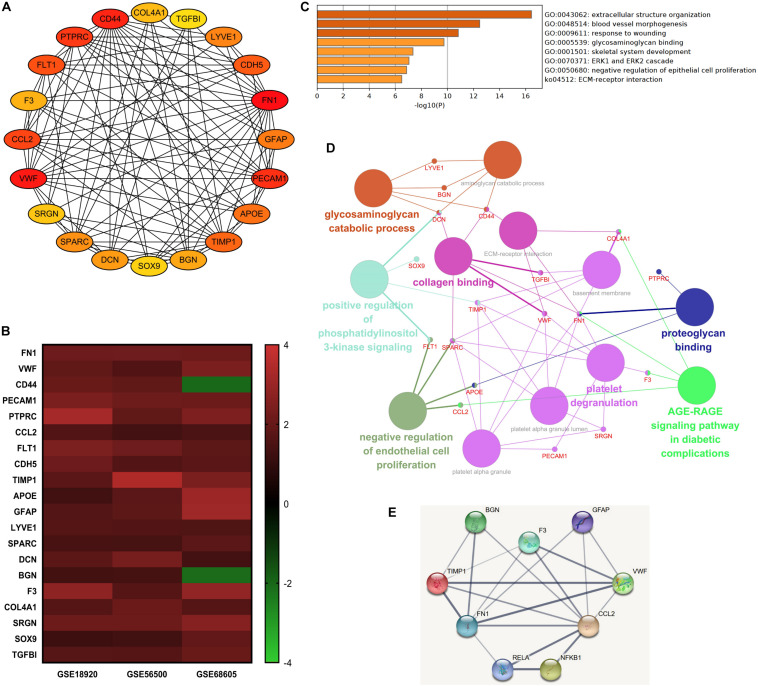
The integrated bioinformatics analysis on hub genes. **(A)** Twenty hub genes were identified by the maximum correlation criterion scoring in Cytoscape. **(B)** The heat map of expression levels of 20 hub genes in the original gene expression series. Red indicated higher expression level in motor neurons in sALS compared with healthy controls, while green indicated lower expression level. **(C)** Enrichment analysis of the identified hub genes by Metascape. **(D)** The visualization of the hub genes and the functional group network constructed by the ClueGO and CluePedia plugin of Cytoscape. **(E)** Interactions between the predicted regulatory transcriptional factors and target genes.

The top 20 nodes scoring the highest in MCC by cytoHubba plugin were identified as hub genes in the network (Figure 4A). Besides, the degrees of these nodes were calculated. These hub genes were mostly upregulated in motor neurons of sALS patients in three gene expression profiles (Figure 4B). The detailed descriptions of these hub genes were shown in Table 2.

**TABLE 2:** Top 20 hub genes of PPI network ranked by MCC method in Cytoscape.

**Rank**	**Name**	**MCC score**	**Degree**	**Description**
1	FN1	25,099	42	Fibronectin 1
2	VWF	21,065	26	von Willebrand factor
3	CD44	21,032	28	CD44 molecule (Indian blood group)
4	PECAM1	20,082	18	Platelet and endothelial cell adhesion molecule 1
5	PTPRC	15,701	23	Protein tyrosine phosphatase receptor type C
6	CCL2	14,630	19	C-C motif chemokine ligand 2
7	FLT1	12,964	14	fms related receptor tyrosine kinase 1
8	CDH5	10,935	14	Cadherin 5
9	TIMP1	8,436	21	TIMP metallopeptidase inhibitor 1
10	APOE	7,474	22	Apolipoprotein E
11	GFAP	5,322	18	Glial fibrillary acidic protein
12	LYVE1	5,066	9	Lymphatic vessel endothelial hyaluronan receptor 1
13	SPARC	4,633	17	Secreted protein acidic and cysteine rich
14	DCN	3,770	13	Decorin
15	BGN	3,219	16	Biglycan
16	F3	2,906	11	Coagulation factor III, tissue factor
17	COL4A1	1,624	13	Collagen type IV alpha 1 chain
18	SRGN	1,468	11	Serglycin
19	SOX9	968	11	SRY-box transcription factor 9
20	TGFBI	889	9	Transforming growth factor beta induced

Gene ontology analysis of hub genes demonstrated significant enrichment in extracellular structure organization, response to wounding, glycosaminoglycan binding, and ECM-receptor interaction. But these hub genes were also associated with blood vessel morphogenesis (Figure 4C). The enrichment analysis of hub genes was similar to previous analysis of DEGs. Figure 4D visualized the association between most of these hub genes and the main enrichment GO terms by establishing a functional group network. Notably, most genes were relevant to ECM-related functions or processes, such as collagen, glycosaminoglycan, and proteoglycan binding and ECM-receptor interaction.

### Regulatory Transcriptional Factors Associated With Hub Genes

Subsequently, the online TRRUST database was applied to demonstrate the regulators of the selected hub genes. The top 10 identified TFs were displayed in Table 3. We identified RelA (transcription factor p65) and NF-κB1 (transcription factor p105/p50) from NF-κB family markedly overlapped with seven genes, including *BGN, CCL2, F3, FN1*, *GFAP*, *TIMP1*, and *VWF*. The overall network of target hub genes and these two significant TFs were visualized in Figure 4E. Other TFs such as NFIC, SP1, and a pair of homologous TWIST1 and TWIST2 of the Twist family were also identified.

**TABLE 3:** Top 10 predicted transcription factors of hub genes by TRRUST.

**Rank**	**Key TF**	**Description**	**Target genes**	***P*-value**	**FDR**
1	RELA	v-rel reticuloendotheliosis viral oncogene homolog A (avian)	BGN, CCL2, F3, FN1, GFAP, TIMP1, VWF	1.59E-08	1.41E-07
2	NFKB1	Nuclear factor of kappa light polypeptide gene enhancer in B-cells 1	BGN, CCL2, F3, FN1, GFAP, TIMP1, VWF	1.66E-08	1.41E-07
3	NFIC	Nuclear factor I/C (CCAAT- binding transcription factor)	CCL2, GFAP, VWF	2.93E-06	1.66E-05
4	TWIST1	Twist basic helix-loop-helix transcription factor 1	CD44, FN1, TIMP1	6.52E-06	2.32E-05
5	SP1	Sp1 transcription factor	APOE, CCL2, CD44, F3, SOX9, TIMP1	6.81E-06	2.32E-05
6	EGR1	Early growth response 1	F3, FLT1, FN1	0.000105	0.000299
7	REL	v-rel reticuloendotheliosis viral oncogene homolog (avian)	CCL2, F3	0.000243	0.000591
8	TWIST2	Twist basic helix-loop-helix transcription factor 2	CD44, FN1	0.000316	0.000671
9	ERG	v-ets erythroblastosis virus E26 oncogene homolog (avian)	CDH5,VWF	0.000369	0.000696
10	STAT3	Signal transducer and activator of transcription 3 (acute-phase response factor)	CCL2, GFAP, TIMP1	0.000433	0.000736

## Discussion

### Integrated Analysis Identifies ECM as the Key Regulator in sALS Motor Neurons

Bioinformatics methods directly performed on ALS motor neurons have been applied in former studies. Rabin et al. (2010) demonstrated the altered cell-matrix adhesion as well as transmembrane and secreted protein in sALS motor neurons in a single microarray analysis. The comparison of ALS patients with *C9ORF72* mutation and sporadic ALS exhibited similar gene expression profiles, and a recent study highlighted the gene expression changes relevant to excitotoxicity between human oculomotor neurons and spinal motor neurons (Wong and Venkatachalam, 2019; Patel and Mathew, 2020). Although bioinformatics analysis provided insights into in-depth molecular studies, higher false positive rate and biased results were inevitable in single microarray analysis, which were due to the heterogeneity of the disease, specimen, sample size, and the platform of different microarrays to be analyzed (Wei et al., 2020). Hence, in the present study, we obtained three gene expression profiles from the public database and performed integrated bioinformatics analysis to discover genes that are differentially expressed in motor neurons from sALS patients and healthy controls. A total of 206 DEGs that are intersected in three microarray series were identified. These DEGs are significantly enriched in two parts of functions or biological processes, blood vessel morphogenesis, and extracellular structure organization. The application of STRING and Cytoscape enabled the exploration and visualization of protein-protein interactive networks, where 20 hub genes and two clustered subnetworks were identified. The two subnetworks were associated with the organization of collagen-containing ECM and blood vessel morphogenesis, respectively. These hub genes were mostly upregulated in motor neurons from sALS patients. The subsequent TF prediction proposed RelA-NF-κB1 (p65-p105/p50) as the main regulatory pathway in the hub genes network.

It was suggested by the above integrated analysis that genes relevant to both blood vessel morphogenesis and ECM were differentially expressed in sALS. But considering the study objects of our research, blood vessel morphogenesis was less likely associated with the biological processes and molecular functions of motor neurons in the spinal cord, as motor neurons were not involved in the composition of blood vessels directly. Hence, we would like to discuss more about the role of ECM and related molecules. FN1, CD44, COL4A1, and VWF were related to ECM-receptor interaction, while FN1, CD44, VWF, DCN, SPARC, and TGFBI were relevant to collagen binding process in the extracellular space. Proteoglycan binding and glycosaminoglycan catabolic process were also ECM related processes. Accounting for over 10% of the total volume of the brain between neurons and glia cells, ECM is essential in maintaining the normal functions of the central nervous system (CNS) (Benarroch, 2015). The basement membrane rich in collagen type IV, fibronectin, and laminin contributes to the integrity of brain-blood barrier (BBB), while other ECM molecules, including hyaluronic acid and multiple proteoglycans, were secreted to form ECM and perineuronal and perisynaptic nets in the extracellular space (Dityatev et al., 2010; Benarroch, 2015). Functional research has also revealed the roles of ECM in neural stem cell behavior, axonal growth, and myelination and synaptogenesis (Barros et al., 2011). Moreover, the cell adhesive network constructed by cell adhesion molecules (CAMs) provides connections between ECM molecules and intracellular components, which offers elaborate structural supports for force transmission, cytoskeletal regulation, and intercellular signaling (Kanchanawong et al., 2010; Edwards and Bix, 2019). It remained unclear whether the upregulation of these ECM related genes was the fundamental pathology of degenerated motor neurons or the secondary alternation due to other factors, but the alternation of gene expression affected the homeostasis of ECM to a certain extent.

### Altered Expression of Various ECM-Related Genes in the PPI Network

Of all the upregulated hub genes enriched in ECM in the cellular component subgroup, *FN1* gene scored the highest in the MCC score and node degrees, indicating strong correlations with other genes in the network. Encoded by *FN1* gene, fibronectin is a kind of glycoprotein abundant in the basement membrane during embryogenesis and pathological angiogenesis in a wide range of tissues (Mongiat et al., 2016; Edwards and Bix, 2019). Fibronectin secreted by proliferating neurons and astrocytes is notably responsive in multiple neurologic disorders (Benarroch, 2015). The upregulation of fibronectin and its receptors, α5β1 and αvβ3 integrins in cerebral ischemia suggested the positive role of angiogenesis (Li et al., 2012). In multiple sclerosis (MS), the secretion of fibronectin was mediated by pro-inflammatory cytokines in a strong inflammatory process, while the aggregation of fibronectin in the white matter impaired the function of oligodendrocytes and remyelination of the lesion in MS (Stoffels et al., 2013; Werkman et al., 2020). In peripheral neuropathies, fibronectin was highly expressed in neuron regenerating sites necessary for Schwann cell differentiation and axonal regrowth, and the decreased amount of fibronectin in non-regenerating neurons was accounted for ECM degradation and leakage from disrupted basement membrane (Previtali et al., 2008). Specifically, the axon regeneration and neurite outgrowth of neurons was supported by the α5β1 integrin of fibronectin (Tonge et al., 2012). These results suggested the potential positive role of fibronectin by secreted into ECM in response to injuries. However, the role of fibronectin in the pathogenesis of ALS was still ambiguous. Fibronectin together with collagen I and III were accumulated in skeletal muscle in symptomatic hSOD1G93A mice, indicating the fibrotic process induced by the transforming growth factor-β (TGF-β) signaling pathway (Gonzalez et al., 2017). In human motor cortex, the upregulated TGF-β system also led to the enhanced expression level of FN1 and collagen IV (Peters et al., 2017). On the contrary, plasma fibronectin was found significantly lower in ALS patients and in negative correlation with duration of the disease (Ono et al., 2000). Nevertheless, the expression of fibronectin in plasma did not directly reflect the neurodegeneration in motor neurons.

Apart from FN1, the upregulated COL4A1 and COL12A1 identified in the cluster network were both genes that encode collagen related substances. COL4A1 encoding collagen IV is large molecules as the main components in the basement membrane scaffold (Yurchenco, 2011). Collagen IV deposition occurred in the perivascular space and the basement membrane in ALS patients (Garbuzova-Davis et al., 2012). Such compensatory process as response to the injury of BBB could probably hinder the drug transport into the CNS (Garbuzova-Davis et al., 2016). Collagen IV, together with laminin and vimentin, were the main components of the glial scar after injuries in the spinal cord (Saxena et al., 2012). The upregulation of collagen IV in the basal laminae correlated with the softening of the tissue, a negative factor for neuronal growth (Moeendarbary et al., 2017). On the other hand, the fibril-associated collagen XII is encoded by COL12A1 gene with two variants (Koch et al., 1995). It interacts with multiple of ECM proteins, mainly collagen I fibrils, and other components such as tenascin-X (Chiquet et al., 2014; Delbaere et al., 2020). Remarkably, the collagen XII functioned as the pro-regenerated factor in navigating the axons through non-neuronal lesion sites through the Wnt/β-catenin pathway (Wehner et al., 2017). The altered collagens expression played dual roles in the pathogenesis of ALS. In the present study, we also found the upregulation of TGFBI protein associated with collagen binding in the pathway analysis. TGFBI protein has high affinity in binding with fibronectin and functions in cell adhesion, migration, inflammation, and wound healing (Lakshminarayanan et al., 2015). Multiple studies have linked the activation of TGF-β system and other proinflammatory cytokines to the overexpression of TGFBI protein (Thapa et al., 2007). Taken together, the upregulation of *FN1*, *COL4A1*, and *TGFBI* genes in our study suggested that abundantly expressed fibronectin and other collagen components were associated with the degeneration of motor neurons and the fibrotic process surrounding motor neurons in the spinal cord, partly through the activation of proinflammatory TGF-β pathway.

Moreover, the upregulation of TIMP1 expression in motor neurons suggested changes in the vital dynamic balance of degradation and remodeling of ECM. This was mainly maintained by the interactions between metalloproteinases (MMPs) and tissue inhibitors of metalloproteinases (TIMPs) (Jackson et al., 2017). MMPs are crucial in the development of CNS and many physiological processes of the adult brain by degrading ECM, but the overactivation of MMPs associated with inflammatory response was discovered in multiple neurologic disorders (Singh et al., 2015; De Stefano and Herrero, 2017). As the most studied member, MMP9 was involved in the muscle degeneration process by enhancing ER stress, and its reduction rescued TDP-43 triggered death of motor neurons (Kaplan et al., 2014; Spiller et al., 2019). The upregulation of MMP9 resulted in the destruction of neurovascular unit in the presymptomatic period in ALS-G93A mice, even prior to the degeneration of motor neurons (Miyazaki et al., 2011). The proteolytic activity of MMPs was inhibited by four types of TIMPs, namely TIMP1–TIMP4 (Lukaszewicz-Zajac et al., 2014; Jackson et al., 2017). TIMP1 is secreted in the soluble form in ECM and plays the role as a strong inhibitor for MMP1, MMP3, MMP7, and MMP9 (Lukaszewicz-Zajac et al., 2014). The elevated TIMP-1 level in acute ischemic stroke patients correlated with poor prognosis, suggesting the possible compensatory mechanism to inhibit excessive MMPs activity in maintaining ECM integrity (Zhong et al., 2019). However, the specific role of TIMP-1 in ALS is still unknown, even though the upregulation of TIMP1 was found in serum, cerebrospinal fluid (CSF), and the spinal cord of ALS patients in several studies (Lim et al., 1996; Fang et al., 2009; Niebroj-Dobosz et al., 2010). It seems that the elevated level of TIMP-1 was insufficient to suppress the degradation of ECM or in a malfunction state, suggesting the role of other MMPs or TIMPs in maintaining the balance. The pathological alternation of homeostasis in ECM was hazardous to the integrity of BBB, and the mechanism is worth further exploration.

### Identification of the Potential Regulatory Pathways in Motor Neurons

According to the list of hub genes, TRRUST suggested the key role of p65 and p105/p50 as the most significant prediction of the transcriptional factors, while the STRING online database demonstrated their direct interactions with seven target genes. The p65 and p105/p50 (including precursor proteins p105 and its mature form p50) are both members of NF-κB family, which is often activated in the presence of DNA damage, reactive oxygen species, and intracellular pathogens (Hayden and Ghosh, 2012). The transactivation domains (TADs) in the C-terminal enables the ability of initiating transcription in p65, while p105/p50 without TADs regulates the transcription processes by binding to TADs-containing members or other subunits (Hayden and Ghosh, 2012). The p65/p50 heterodimer is commonly regulated by IκB kinase in the NF-κB pathway (Siggers et al., 2011; Hayden and Ghosh, 2012). Recent studies revealed the complex roles of the activation of NF-κB pathway in the pathogenesis of ALS. Astrocyte-derived NF-κB activation was neuroprotective in presymptomatic stage by delaying disease onset, but it facilitated the inflammatory process by the activation of microglial, which induced motor neuron death in ALS models (Frakes et al., 2014; Ouali Alami et al., 2018). NF-κB activation in motor neurons resulted in the pathological TDP-43 aggregation in cytoplasm and nucleus as the landmark pathology in most ALS cases (Swarup et al., 2011; Picher-Martel et al., 2015). Recent studies demonstrated the fibrosis mediated by NF-κB activation in multiple diseases including cardiac hypertrophy and idiopathic pulmonary fibrosis, and the inhibitions of such pathways prevented excessive ECM deposition and remodeling (Kumar et al., 2011; Song et al., 2016). In spinal cord injury, the fibrotic scarring produced by the chronic fibrotic response limited the axons recovery in the lesion site and motor functional recovery of the mouse model, which can be reversed by eliminating the isoform of fibronectin containing the Extra Domain A (FnEDA) (Cooper et al., 2018). Similarly, FnEDA also exacerbated stroke outcome through initiating inflammation (Dhanesha et al., 2019).

On the other hand, C-C motif chemokine ligand 2 (CCL2) was also correlated with the NF-κB pathway directly in the interaction network. Also known as monocyte chemoattractant proteins 1 (MCP1), CCL2 is widely expressed in CNS and associated with the neuroinflammation process by binding to its receptor CCR2 (Conductier et al., 2010). It was previously confirmed that CCL2 (MCP1) was upregulated in serum, CSF, and peripheral blood mononuclear cell in ALS patients (Kuhle et al., 2009; Gupta et al., 2011a,b). Specifically, an early innate immune response represented by MCP1-CCR2 interaction was identified in motor cortex in mouse model, where MCP-1 were found in microglia-like cells (Jara et al., 2017). In a further research concerning motor cortex of ALS with TDP-43 pathology, the CCL2 (MCP1) expression increased in Betz cells, which was even prior to the infiltration of CCR2 positive monocytes (Jara et al., 2019). These studies were significant in proposing direct evidence for MCP1-CCR2 interaction in neuroinflammation in the motor cortex. CCL2 (MCP1) was also abundantly expressed in motor neurons in the spinal cord, and its level elevated during the disease progression in ALS mice (Kawaguchi-Niida et al., 2013). Considering the overexpression of CCL2 among the three analyzed gene expression profiles included in our study, it represented the upregulated neuroinflammation process surrounding motor neurons in sALS.

Taken together, we proposed an interaction network with several hub genes and the indicated regulatory pathways, and suggested that the dysregulation of NF-κB pathway induced by neuroinflammation contributes to the overexpression of fibronectin and a series of fibrotic processes as the major changes in the spinal cord, which could be the potential target for ALS therapy.

### Limitations of the Study and Conclusion

To avoid biased results in bioinformatics analysis, we included multiple gene expression profiles of motor neurons from sALS patients and healthy controls for analysis, aiming to provide insights of the genetic patterns of the motor neurons in the advanced disease state. Nevertheless, there are still some limitations in our study. First, as the analysis on gene expression profiles in advanced state of motor neurons did not adequately explain the progression of the disease, it is still necessary to elucidate whether the upregulation of the hub genes are primary or secondary changes in the pathogenesis of sALS. Second, due to the lack of clinical data of these sALS patients, we are not able to perform survival analysis or demonstrate the association of hub genes and disease progression in sALS patients. More importantly, although we pointed out multiple upregulated molecules and related pathways in motor neurons in terminal-stage sALS, we suggest that validation from cellular, animal experiments, and even larger cohort are needed in demonstrating the overall alternation in ECM molecules during the disease progression.

In this study, the integrated bioinformatics analyses revealed the upregulation of multiple genes related to biological processes of ECM components and proposed Rela/NF-κB1 as the regulatory transcriptional factors in the pathogenesis of sALS. The bioinformatics analysis on alternation of gene expression profiles and their protein interaction networks will not only enhance our understanding of sALS but also provide insights in searching for distinct biomarkers and therapies.

## Data Availability Statement

All datasets generated for this study are included in the article/supplementary material, further inquiries can be directed to the corresponding author.

## Author Contributions

JL and XY contributed to the study design and management. JL and PH contributed to the data collection. PH, WC, CY, and HS contributed to the data analysis and visualization. JL contributed to writing the manuscript. HS and XY contributed to the manuscript review and editing. All authors reviewed and approved the final manuscript.

## Conflict of Interest

The authors declare that the research was conducted in the absence of any commercial or financial relationships that could be construed as a potential conflict of interest.

## Footnotes

1 https://www.ncbi.nlm.nih.gov/geo/

2 https://www.ncbi.nlm.nih.gov/geo/geo2r/

3 http://www.bioconductor.org/

4 http://bioinformatics.psb.ugent.be/webtools/Venn/

5 https://metascape.org/

6 https://string-db.org/

7 https://cytoscape.org/index.html

8 https://www.grnpedia.org/trrust/
